# Viral Etiology of Encephalitis in Children in Southern Vietnam: Results of a One-Year Prospective Descriptive Study

**DOI:** 10.1371/journal.pntd.0000854

**Published:** 2010-10-26

**Authors:** Le Van Tan, Phan Tu Qui, Do Quang Ha, Nguyen Bach Hue, Lam Quoi Bao, Bach Van Cam, Truong Huu Khanh, Tran Tinh Hien, Nguyen Van Vinh Chau, Tran Tan Tram, Vo Minh Hien, Tran Vu Thieu Nga, Constance Schultsz, Jeremy Farrar, H. Rogier van Doorn, Menno D. de Jong

**Affiliations:** 1 Oxford University Clinical Research Unit, South East Asia Infectious Diseases Clinical Research Network, Hospital for Tropical Diseases, Ho Chi Minh City, Vietnam; 2 Hospital for Tropical Diseases, Ho Chi Minh City, Vietnam; 3 Paediatric Hospital Number One, Ho Chi Minh City, Vietnam; 4 Centre for Tropical Medicine, Oxford University, Oxford, United Kingdom; 5 Centre for Poverty-Related Communicable Diseases, Academic Medical Center, University of Amsterdam, Amsterdam, The Netherlands; 6 Department of Medical Microbiology, Academic Medical Center, University of Amsterdam, Amsterdam, The Netherlands; Centre for Cellular and Molecular Biology (CCMB), India

## Abstract

**Background:**

Acute encephalitis is an important and severe disease in children in Vietnam. However, little is known about the etiology while such knowledge is essential for optimal prevention and treatment. To identify viral causes of encephalitis, in 2004 we conducted a one-year descriptive study at Children's Hospital Number One, a referral hospital for children in southern Vietnam including Ho Chi Minh City.

**Methodology/Principal Findings:**

Children less than 16 years of age presenting with acute encephalitis of presumed viral etiology were enrolled. Diagnostic efforts included viral culture, serology and real time (RT)-PCRs. A confirmed or probable viral causative agent was established in 41% of 194 enrolled patients. The most commonly diagnosed causative agent was Japanese encephalitis virus (n = 50, 26%), followed by enteroviruses (n = 18, 9.3%), dengue virus (n = 9, 4.6%), herpes simplex virus (n = 1), cytomegalovirus (n = 1) and influenza A virus (n = 1). Fifty-seven (29%) children died acutely. Fatal outcome was independently associated with patient age and Glasgow Coma Scale (GCS) on admission.

**Conclusions/Significance:**

Acute encephalitis in children in southern Vietnam is associated with high mortality. Although the etiology remains unknown in a majority of the patients, the result from the present study may be useful for future design of treatment and prevention strategies of the disease. The recognition of GCS and age as predictive factors may be helpful for clinicians in managing the patient.

## Author Summary

Viral encephalitis is associated with high morbidity and mortality in Vietnam. However little is known about the causes of the disease due to a lack of diagnostic facilities in this relatively resource-poor setting. Knowledge about the etiologies and clinical outcome of viral encephalitis is necessary for future design of intervention studies targeted at improvement of clinical management, treatment and prevention of the disease. We report the viral agents, clinical outcome and prognostic factors of mortality of encephalitis in children admitted to a referral hospital for children in southern Vietnam. We show that about one third of the enrolled patients die acutely, and that mortality is independently associated with patient age and Glasgow Coma Scale on admission. Japanese encephalitis, dengue virus and enterovirus (including enterovirus 71) are the major viruses detected in our patients. However, more than half of the patients remain undiagnosed, while mortality in this group is as high as in the diagnosed group. This study will benefit clinicians and public health in terms of clinical management and prevention of childhood encephalitis in Vietnam.

## Introduction

Acute encephalitis is associated with high morbidity and mortality and affects both children and adults. There have been few population-based studies, reporting incidences ranging between 3.5 and 7.4 cases per 100.000 patient-years [1]. Viruses are regarded as the most important etiological agents of encephalitis worldwide. However, in the majority of cases a specific infectious etiology cannot be found. Furthermore, the specific causes of the illness show considerable geographic and age dependent variation. In a population-based study in the United Kingdom, herpes simplex virus (HSV) was the most common virus diagnosed, and the proportion of cases with an identified etiology was significantly lower in children (33%) than in adults (45%) [2]. In the California Encephalitis Project, a confirmed or probable infectious etiology was found in only 16% of cases, with HSV-1 most commonly found in adult patients and enteroviruses in children [3].

In Southeast Asian countries like Cambodia and Vietnam, Japanese encephalitis virus (JEV) has been the leading reported cause of acute encephalitis in children, accounting for 31% to 45% of cases [4], [5]. However, the causes of the disease in the remaining cases were not extensively studied. Improved insight into the specific viral etiology and pathogen-specific clinical outcome of acute encephalitis in this region is essential to guide strategies for prevention and clinical management. We conducted a one-year prospective descriptive study of children with acute encephalitis who were admitted to a pediatric referral hospital in Ho Chi Minh City, Vietnam.

## Methods

### Study site

The Childrens's Hospital Number One (CH1) in Ho Chi Minh City is an 850-bed pediatric referral hospital for the southern provinces of Vietnam. Each year, the hospital admits between 400–800 patients less than 16 years of age with acute symptoms of fever and altered consciousness (including seizures). Of these patients, approximately 40% are considered to have acute encephalitis of viral origin based on clinical judgment of pediatricians at CH1.

### Patients

Children admitted between January 1 and 31 December 2004 with suspected acute encephalitis of viral origin, based on the clinical judgment of admitting physicians, and with no preexisting neurological conditions or evidence of bacterial meningitis by microscopy or culture of cerebrospinal fluid (CSF) samples, and no febrile convulsion (defined by a single convulsion lasting less than 15 minutes with regaining of consciousness within 60 minutes in a child between 6 months and 6 years of age) were eligible for inclusion in the study after provision of written informed consent by the patient's parents or legal guardians. Tests of human immunodeficiency virus and tuberculosis infections were not performed when recruiting the patients.

### Clinical data and specimen collection

Detailed demographic and clinical data, including routine blood and CSF hematology and chemistry laboratory investigations, were collected on case record forms at enrollment and during follow-up. Clinical outcomes were defined as death, full recovery, and severe, moderate or minor neurological sequelae, defined based on neurological examination, degree of independent functioning and controllability of seizures. For etiological investigations, acute and convalescent CSF, plasma and serum samples were obtained at enrollment and around 2 weeks later or at discharge. In addition, throat and rectal swabs were collected in viral transport medium (VTM) at enrollment.

### Etiological laboratory investigations

#### Nucleic acid extraction

Total nucleic acid was isolated from 180µL of clinical specimens (CSF, plasma, VTM) using a silica-guanidinium thiocyanate-based procedure [6], [7], recovered in 100µL of TE (10 mM Tris-HCl, 1 mM EDTA, pH 8.0, Sigma-Aldrich Pte. Ltd., Singapore) and stored at −80°C until used. For detection of human parechoviruses, enterovirus in blood, and bacterial pathogens, nucleic acids were isolated with the automated easyMAG system (BioMérieux, Marcy l'Étoile, France), following the manufacturer's instructions.

#### Bacterial investigations

Available CSF samples of enrolled patients were retrospectively analyzed for bacterial infections using internally controlled real-time polymerase chain reaction (PCR) methods for detection of 4 bacterial pathogens that are most frequently associated with meningitis in Vietnam: *Streptococcus pneumoniae*, *Haemophilus influenzae* type b (Hib), *Neisseria meningitidis*, and *Streptococcus suis* [8], [9]. Primer sequences and probes are listed in table 1. All oligonucleotides were ordered from Sigma-Proligo (Singapore)

**Table 1:** Oligonucleotide sequences of primers and probes used in this study.

Pathogen	Gene target	Sample tested	Oligo sequence (5′ – 3′)					Number of PCR samples			
			Forward	Reverse	probe	Reference	Internal control	CSF	Throat swab	Rectal swab	plasma
**HSV** €	gB	a	CCG TCA GCA CCT TCA TCG A	CGC TGG ACC TCC GTG TAG TC	6-FAM-CCA CGA GAT CAA GGA CAG CGG CC-TAMRA	[36]	Y	192	ND	ND	ND
**Enteroviruses** ¥	5′UTR	a, b, c, d±	CCC TGA ATG CGG CTA AT	ATT GTC ACC ATA AGC AGC C	6-FAM-CGG AAC CGA CTA CTT TGG GT-TAMRA	[37]	Y	192	177	178	9
**Enterovirus 71**	VP1/VP3	a, b, c	ATAATAGCAYTRGCGGCAGCCCA	AGAGGGAGRTCTATCTCYCC	−	[38]	ND	3	23	37	ND
**Parechoviruses**	5′UTR	a	CTGGGGCCAAAAGCCA	GGTACCTTCTGGGCATCCTTC	6-FAM-AAACACTAGTTGTA(A/T)GGCCC-NFQ	[31], [39]	ND	162	ND	ND	ND
**DENV-1**	3′UTR	a, d, e	ATCCATGCCCATCAYCAATG	GATCARTGGTGTGGATCCCTG	6-FAM-TCAGTGTGGAATAGGGTTTGGATAGAGGAA-BHQ-1	[26]	Y	16	ND	ND	16
**DENV-2**	3′URT	a, d, e	ACAAGTCGAACAACCTGGTCCAT	GAGAAGACCAATGGTGCGGC	6-FAM-GTT+T+Tg+T+CT+TC+CA+TCCA-BHQ-1		Y	16	ND	ND	16
**DENV-3**	3′UTR	a, d, e	TTTCTGCTCCCACCACTTTCAT	AGACTRGCATCCAACGCCA	6-FAM-AAGAAAGTTGGTAGTTCCCTGCAGACCCCA-BHQ-1		Y	16	ND	ND	16
**DENV-4**	3′UTR	a, d, e	GYGTGGTGAAGCCYCTRGAT	ATGAAGGATGGCCGYTCACT	6-FAM-ACTTCCCTCCTCTTYTTGAACGACATGGGA-BHQ-1		Y	16	ND	ND	16
***Influenzavirus A***	M	a	GACAAGACCAATCCTGTCACYTCTG	AAGCGTCTACGCTGCAGTC	6-FAM-TTCACGCTCACCGTGCCCAGTGAGC-TAMRA	[12]	ND	191	178	ND	ND
**EAV**	NSP	NA	CATCTCTTGCTTTGCTCCTTA	AGCCGCACCTTCACATTG	Cy5-GCGCTCGCTGTCAGAACAACATTATTGCCCACAGCGCG-BHQ-3	[26]	NA	NA	NA	NA	NA
**PhHV**	gB polymerase	NA	GGG CGA ATC ACA GAT TGA ATC	GCG GTT CCA AAC GTA CCA A	Cy5-TTT TTA TGT GTC CGC CAC CAT CTG GAT C′-BHQ3	[8]	NA	NA	NA	NA	NA
**MTV/SFV_1** *	E	a	TATTGGCTAAAGGAAAAAGGGACAG	CATAGCGACACCACAAAAGAAGG	−		ND	192	ND	ND	ND
**MTV/SFV_2** *	E	a	TACTGGCTAAAAGAAAAAGGAACTG	CACAGGGACACCACGAAGGATGG	−				ND	ND	ND
**Flavivirus**	NS5	a	AACATGATGGGiAARAGAGARAA	GTGTCCCAiCCiGCiGTRTCATCiGC	−	[40]	ND	192	ND	ND	ND
**CMV**	IE	a	CCAAGCGGCCTCTGATAACCAA	GGTCATCCACACTAGGAGAGCAGAC	−	[41]	ND	192	ND	ND	ND
***N. meningitides***	*ctrAe*	a	GCTGCGGTAGGTGGTTCAA	TTGTCGCGGATTTGCAACTA	6-FAM-CATTGCCACGTGTCAGCTGCACAT-TAMRA	[9]	Y	162	ND	ND	ND
***H. influenzae*** ** type B**	*bexA*	a	GGCGAAATGGTGCTGGTAA	GGCCAAGAGATACTCATAGAACGTT	6-FAM-CACCACTCATCAAACGAATGAGCGTGG-TAMRA		Y	162	ND	ND	ND
***S. pneumoniae***	*ply*	a	TGCAGAGCGTCCTTTGGTCTAT	CTCTTACTCGTGGTTTCCAACTTGA	6-FAM-TGGCGCCCATAAGCAACACTCGAA-TAMRA		Y	162	ND	ND	ND
***S. suis***	*cps2J*	a	GGTTACTTGCTACTTTTGATGGAAATT	CGCACCTCTTTTATCTCTTCCAA	6-FAM-TCAAGAATCTGAGCTGCAAAAGTGTCAAATTGA-TAMRA	[8]	Y	162	ND	ND	ND

Notes:

*in house, a: CSF, b: throat swab, c: rectal swab, d: plasma, e: culture supernatant, ND: not done, NA: non applicable, EAV: equine arteritis virus, PhHV: phocid herpesvirus, NFQ: non-fluorescence quencher.

€ Due to the availability of the assay, a commercial SYBR Green PCR master mix (Bio-Rad, USA) was used for the detection of HSV DNA when the study was started and was then replaced by a HSV Taqman real-time PCR.

**±:** only if detected by RT-PCR in the other sample types.

**¥:** internal control was not applied for detection of enterovirus in CSF samples.

CSF serology and virus culture of CSF and sera were done in all 194 enrollees.

#### PCR and RT-PCR analyses for viral infections

Conventional or real-time reverse transcription (RT) PCRs were used to detect HSV-1 and 2, cytomegalovirus, enteroviruses (generic), enterovirus 71, influenza A virus, Me Tri virus/Semliki Forest virus [10], human parechoviruses, flaviviruses (generic), and dengue viruses (DENV) types 1 to 4 if serology and/or virus culture results were suggestive of dengue. Primer and probe sequences are listed in Table 1 and were all adapted from previous publications except for those targeted at Me Tri/Semliki Forest virus which were newly designed [10].

#### Serology

A capture IgM ELISA assay (Venture Technologies, Sarawak, Malaysia) that utilizes inactivated antigens from JEV and DENV1 – DENV4 was used to detect and distinguish between JEV-specific and DENV-specific IgM antibodies in CSF specimens, and was performed as previously described [11]

#### Virus isolation

Acute CSF and serum specimens were inoculated onto C6/36 mosquito cells, RD (human rhabdomyosarcoma cells), BHK-21 (baby hamster kidney cells) and African green monkey kidney (Vero) cells (ECACC, Wiltshire, UK). Cells were observed daily for cytopathic effect and viruses were identified using standard procedures of direct- and indirect immunofluorescence staining assays, and virus-specific (RT) PCRs.

### Diagnostic interpretation

Depending on clinical sample types in which evident of viral infetion was identified, the encephalitis etiology was considered as definitive, probable or possible cause. Details on the diagnostic interpretation are presented in table 2.

**Table 2:** Diagnostic interpretation.

Category	Criteria	Virus
**Definitive**	Viral detection by PCR or culture in CSF, or detection of viral specific IgM by serologic test in CSF	JEV, DENV, EV, influenza A, CMV and HSV
	Viral detection in both rectal and throat swabs as well as in blood by PCR, ANDAbsence of other viruses detected in CSF	EV
**Probable**	Viral detection in both rectal and throat swabs by PCR, ANDAbsence of other viruses detected in CSF	EV
**Possible**	Viral detection in either rectal or throat swab by PCR, ANDAbsence of other viruses detected in CSF	EV and influenza A
	Viral-specific IgM detected in blood by serologic test or viral RNA detected in plasma by PCR, ANDAbsence of other viruses detected in CSF	DENV

### Statistical analysis

Chi-square test, Fisher exact test, independent samples t test and Wilcoxon rank-sum test were used to compare data between groups of patients when appropriate by using either SPSS for Windows version 14 (SPSS Inc, Chicago, USA) or statistical software R version 2.9.0 (http://www.r-project.org). In addition, to analyze potential prognostic factors for fatal outcome, univariate analyses (including linear regression) and logistic regression were applied.

### Ethical approval

This study was approved by the Institutional Review Board of CH1 and the OXford TRopical Ethics Committee (OXTREC), University of Oxford, UK. Written informed consent was obtained from a parent or guardian of each enrolled patients.

## Results

### Patients

A total of 194 patients with a clinical syndrome of acute encephalitis of presumed viral etiology were enrolled between January 1 and December 31, 2004.

Details of clinical features and outcome of enrolled patients are presented in Table 3. Overall mortality was observed in 57/194 (29%) of the patient, while 48/194 (25%) had neurological sequelae at discharge, including: severe sequelae in 10%, moderate in 10% and mild in 5% (Table 3).

**Table 3:** Characteristics of enrollees and comparisons between patient groups.

		Total enrollees	Bacterial PCR results				Fit the predefined case definition			
			Negative (n = 151)	Positive (n = 12)	*P* value	OR (95% CI)	Yes (n = 132)	No (n = 62)	*P* value	OR (95%CI)
**Demographics**	Male	124(64)	94 (62)	7 (58)	0.7	ND	80 (61)	44 (71)	0.16	ND
	Age (median; IQR)	3 (1–7)	3 (1–7)	1 (0–6.5)	0.16	ND	3 (1–7)	2 (1–6)	0.13	NA
	Non-HCMC resident	139 (72)	107 (71)	10 (83)	0.5	ND	102 (77)	37 (60)	0.011	2.3 (1.2–4.4)
	Referral#	133 (69)	100 (62)	11 (92)	0.1	ND	95 (72)	38 (61)	0.133	ND
**Hospitalization** ≠		9 (6–14)	8 (6–13)	9.5 (6–27)	0.3	ND	9 (6–13)	8.5 (6–18)	0.63	NA
**Clinical symptoms**	History of fever	181 (93)	139 (96)	11 (92)	0.4	ND	128 (100)	53 (90)	0.001	3.4 (2.7–4.28)
	Fever*	142 (73)	105 (70)	10 (83)	0.5	ND	99 (76)	43 (69)	0.36	ND
	History of convulsion	144 (74)	110 (73)	11 (92)	0.2	ND	100 (76)	44 (71)	0.47	ND
	Convulsions	31 (16)	20 (13.6)	4 (33)	0.09	ND	24 (19)	7 (12)	0.19	ND
	Limb weakness	60 (31)	49 (33)	3 (25)	1.0	ND	50 (39)	10 (16)	0.002	3.27 (1.5–7.0)
	Neck stiffness	44 (23)	35 (23)	3 (27)	0.7	ND	38 (29)	6 (10)	0.003	3.75 (1.5–9.4)
	Skin Rash	13 (7)	8 (5.3)	0	1.0	ND	7 (5)	6 (10)	0.35	ND
	GCS≤9	123 (63)	94 (62)	8 (67)	1.0	ND	91 (69)	32 (52)	0.019	2.08(1.1–3.9)
**JEV vaccination**	Total	36 (19)	27 (19)	2 (18)	0.8	ND	24 (19)	12 (20)	0.89	ND
	Complete	24 (12)	19 (13)	1 (9)	ND	NA	16 (13)	8(13)	ND	NA
	Incomplete	12 (6)	8 (6)	1 (9)	ND	NA	8 (6)	4 (7)	ND	NA
**Outcome**	Death	57 (29)	43 (29)	4 (33)	0.6	NA	40 (30)	17 (27)	0.015	NA
	Severe sequelae	20 (10)	14 (9)	2 (17)			11 (8)	9 (15)		
	Moderate sequelae	19 (10)	16 (11)	0			18 (14)	1 (1.6)		
	Mild sequelae	9 (5)	7 (5)	0			8 (6)	1 (1.6)		
	Full recovery	89 (46)	71 (47)	6 (50)			55 (41)	34 (55)		
**CSF laboratory**	CSF WBC count$	2 (1–51)	2 (1–43)	98 (17–741)	0.001	ND	8 (1–63)	2 (1–3)	<0.001	NA
	CSF pleocytosis	83 (43)	60 (40)	9 (82)	0.01	0.15 (0.03–0.7)	71 (54)	12 (20)	<0.001	4.73 (2.30–9.7)

NOTE: Data are no. (%) of patients; denominators may vary slightly; IQR = interquartile range,

$ median, cells/mm^3^, IQR,

*Fever at admission (≥37.5°C),

# referred from other hospitals,

**≠:** day, median, IQR; GCS, Glasgow Coma Scale.

### Pathogens

#### PCR detection of bacterial pathogens

In total, CSFs from 163 patients were available for bacterial PCR investigation. Evidence of bacterial infection was found in 12 (6%) patients, including *Haemophilus influenzae* type b in 6 patients and *Streptococcus pneumoniae* in another 6 patients. Evidence of viral co-infection was found in CSF samples of 3 patients (*Haemophilus influenzae* with EV or JEV in 2 patients, and *Streptococcus pneumoniae* with JEV in 1 patient). Except for CSF cell counts, no difference in patient characteristics and outcome between patients with negative and positive bacterial PCR was observed (Table 3).

#### Definitive and probable viral etiologies

A definitive or probable viral etiology was identified in 80 of 194 (41%) enrolled patients (Table 4 and Table 5). The most frequently identified etiologies were definitive JEV infection (50 cases, 26%), followed by enterovirus (18 cases, 9.3%), and definitive dengue viruses (9 cases, 4.6%). Of the enteroviral infections, only 4 of 18 were considered definitive encephalitis based on viral detection in CSF or blood. HSV, CMV and influenza A virus were detected in CSF of 1 patient each. The case of influenza encephalitis was caused by highly pathogenic avian influenza A (H5N1) virus and has previously been described [12].

**Table 4:** Laboratory results of three most common viruses (JEV, DENV and EV) detected in this study.

Virus	Clinical samples	Detected by			Diagnostic category	Total (%)
		Serology	PCR	Culture		
JEV	CSF	50	0	0	Definitive	50 (26)
DENV	CSF	7	4	6	Definitive	9 (4.6)
EV	CSF	ND*	3	0	Definitive	4 (2.1)
	Rectal, throat and blood	ND	1	ND		
	Rectal and throat	ND	14	ND	Probable	14 (7.2)

NOTE:

*ND: not done.

**Table 5:** Viral etiology of encephalitis patients and comparisons of patient characteristics.

		*JEV	*DENV	EV					*HSV	*Inf. A	*CMV	*Diag.	*Undiag.	Diag. versus Undiag.	
				EV		EV71		Total						*P* value	OR (95%CI)
				Definitive	probable	Definitive	probable								
**Diagnosis in**		50 (26)	9 (4.6)	2	10 (5.1)	2	4 (2.1)	18 (9.3)	1	1	1	80 (41)	114 (59)	NA	NA
**Outcome**	Death	8 (16)	1 (11)	2	4 (40)	0	3 (75)	9 (50)	0	1	0	19 (24)	38 (33)	0.037	ND
	Severe sequelae	5 (10)	1 (11)	0	2 (20)	0	0	2 (11)	0	0	1	9 (11)	11 (10)		
	Moderate sequelae	11 (22)	0	0	2 (20)	1	0	3 (17)	0	0	0	14 (18)	4 (4)		
	Minor sequelae	3 (6)	0	0	0	1	0	1 (6)	0	0	0	4 (5)	5 (4)		
**Characteristics**	Age	6 (3–10)	8 (0–13)	1 &13	1.2 (0.75–3.2)	<1& 2	1.2 (1–2.6)	1.3 (0.75–3.3)	4	4	0	4 (1–9)	2 (1–6)	0.002	ND
	Male	27 (54)	8 (89)	2	10 (100)	1	3 (75)	16 (89)	1	1	1	54 (68)	70(61)	0.3	ND
	Non-HCMC resident	43 (86)	7 (78)	2	8 (80)	1	3 (75)	14 (78)	1	1	1	67 (84)	72 (63)	0.002	3 (1.5–6.1)
	Referral	39 (78)	8 (89)	1	7 (70)	1	1 (25)	10 (56)	NA	1	1	60 (75)	73 (63)	0.1	ND
	History of fever	48 (100)	9 (100)	2	9 (90)	2	4 (100)	17 (100)	1	1	1	76 (100)	105 (92)	0.08	ND
	Fever	37 (75.5)	6 (67	2	8 (80)	1	3 (75)	14 (78)	0	1	0	58 (73)	84 (74)	0.9	ND
	Limb weakness	18 (37)	2 (22)	1	3 (30)	1	3 (75)	8 (44)	1	0	0	29 (37)	31 (27)	0.2	NA
	Neck stiffness	19 (38)	2 (22)	1	1 (10)	0	0	2 (11)	0	0	1	23 (29)	21 (18)	0.08	ND
	History of convulsion	36 (72)	6 (67)	2	5 (50)	0	2 (50)	9 (50)	1	1	1	54 (68)	90 (79)	0.07	NA
	Convulsion	3 (6.3)	1 (11)	0	2 (20)	0	0	2 (11)	0	0	1	7 (9)	24 (21)	0.03	2.7(1.1–6.7)
	GCS (≤9)	32 (64)	3 (33)	1	7 (70)	1	3 (75)	12 (67)	0	1	1	49 (61)	74 (65)	0.6	ND
	White cells	51.5 (20–102)	2 (1–2.5)	61 & 108	13.5 (1.5–96)	22&164	59 (11–102)	22 (4.3–99)	0	1	NA	41 (2–92)	0(1–5)	<0.001	ND
	CSF pleocytosis	40 (80)	0	2	7 (70)	2	3 (75)	14 (78)	0	0	NA	54 (68)	29 (25)	<0.001	6.9 (3.61–13.1)
	Hospital stay	9 (7–15)	8 (7–12)	2–8	3.5 (2–14)	8–28	1.5 (1–7)	6 (2–14.3)	6	3	30	9(6–14)	9 (5–15)	0.7	ND

NOTE: Data are no. (%) of patients; denominators may vary.

For continuous variables: data are presented as median (IQR); NA, non- applicable; ND, not done; OR, odds ratio; CI, confident interval; undiag., undiagnosed group; Diag., diagnosed group.

Among the patients with definitive etiological diagnoses, infections with additional viruses in single non-CNS samples (rectal or throat swab) were detected in 14 patients including EV (n = 8) or influenza A virus (n = 2) in patients with definitive JE, EV (n = 3) in patients with definitive DENV encephalitis, and influenza A virus in a patient with HSV encephalitis.

Characteristics and outcomes of patients with definitive or probable viral etiologies are shown in Table 5. The case fatality proportion was significantly higher in patients with enteroviral infection than in those with JEV infection: 9/18 (50%) versus 8/50 (16%) respectively (OR, 5.3; 95% CI, 1.6–17.3; *P* = 0.009). Neurological sequelae were common in both groups (Table 5). Patients with enteroviral encephalitis were significantly younger than Japanese encephalitis patients (median; IQR: 1.3; 0.75–3.3 years versus 6; 3–10 years respectively; *P*<0.001).

#### Possible- and unknown etiologies

A possible viral etiology was detected in 27/194 (14%) of patients, including enteroviruses in 11/194 patients (5.7%), dengue in 7/194 patients (3.6%), influenza A virus in 6/194 patients (3%), combined EV and dengue in 1 patient, and combined EV and influenza A virus in 2 patients. In 87/194 (45%) patients no pathogen could be found or implicated in any specimen.

As comparison found not statistical difference in clinical outcomes and patient characteristics between patients with possible viral diagnoses and those with no diagnoses (data not shown), clinical data of these two groups (undiagnosed group) were combined and compared against patient group of definitive or probable viral diagnosis (diagnosed group). A higher mortality was observed in the undiagnosed group (24% vs 33%, *P* = 0.037, Table 5). Patients with undiagnosed illness were younger, more likely to live in Ho Chi Minh City, more likely to present with convulsion, less likely to have CSF pleocytosis, and had lower CSF white blood cell counts (Table 5).

### Temporal distribution of etiologies

Patients were enrolled throughout the year. JE occurred throughout the year but there was a peak in March and during the months of June and July consistent with the rainy season. No clear seasonality was observed for other etiologies but numbers were small (data not shown).

### CSF PCR and serology in relation to illness day at admission

**Figure 1:**
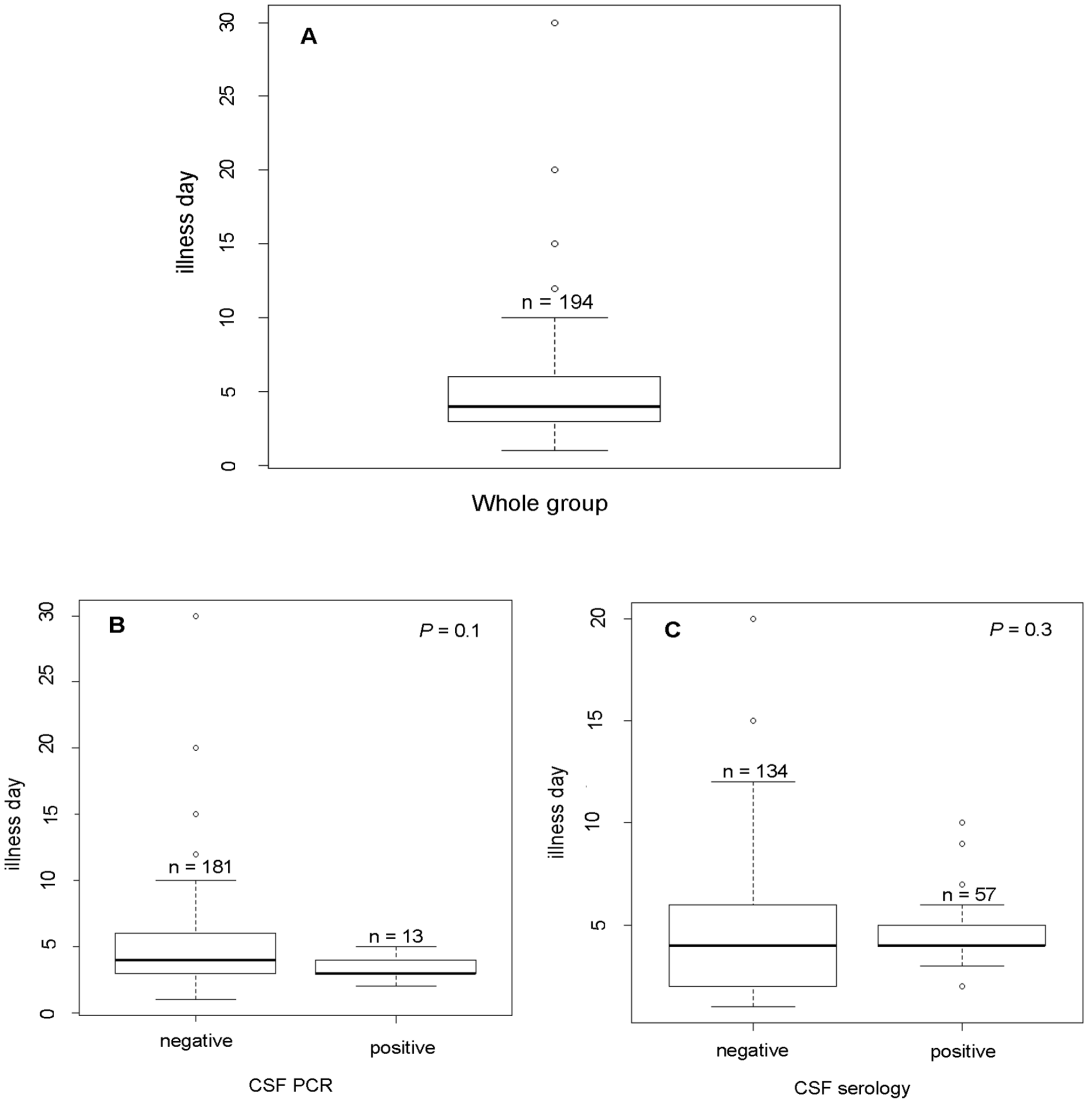
Illness day on admission Vs CSF PCR and serology. A, whole group; B, comparison of illness day on admission of viral PCR negative and viral PCR negative group; C, CSF serology negative and CSF serology positive groups.

Since detection of virus or antibodies may differentially be affected by the timing of specimen collection, we analysed diagnostic yields in CSF specimens by PCR and serology in relation to illness day at the time of sampling (Figure 1). Overall, the median illness day at admission was 4 days (IQR: 3–6). No significant differences in illness days at specimen collection were observed between patients with detectable virus or IgM in CSF and those with negative diagnostic results. However, there was a trend towards a shorter illness duration at time of sampling in PCR positive patients (median, IQR: 3.5, 3–4 versus 4.8, 3–6, *P* = 0.1) (Figure 1).

### Prognostic factors for fatal outcome

Of the 57 fatal cases, 28 patients (49%) died within 7 days after onset of illness (Table 6). Twenty-nine (51%) of fatal cases were between 0 and 1 years of age compared to 43 of 137 (31%) surviving patients. Univariate analyses were used to screen for potential factors associated with fatal outcome (Table 7), and demonstrated that the occurrence of convulsions on admission, the presence of limb weakness, GCS, and age were all significantly associated with fatal outcome, whereas illness day on admission, history of convulsion, and gender were not (Table 7). However, only GCS and age remained independently associated risk factors for fatal outcome in logistical regression analysis (Table 7).

**Table 6:** Time (week) to death since onset of illness.

	Time (week)			
	1	2	3	Total
Total	28 (49)	16 (28)	13 (23)	57
JEV	2 (3.5)	3 (5)	3 (5)	8 (14)
EV	6 (10.5)	2 (3.5)	1 (2)	9 (16)
DENV	1 (1.8)	0	0	1 (2)
Influenza A	0	1 (2)	0	1 (2)
Undiagnosed	19 (33)	10 (17.5)	9 (16)	38 (67)

NOTE: data are no. (% of total) fatal patients.

**Table 7:** Univariate and adjusted logistic regression to determine prognostic factors of fatal outcome.

*Factors	Univariate			Adjusted#		
	OR	95% CI	*P*-value	OR	95% CI	*P*-value
Age (by +1 year)	0.91	0.84–0.99	0.047	0.89	0.80–0.99	0.038
GCS (by +1)	0.62	0.52–0.72	<0.001	0.60	0.51–0.71	<0.001
Convulsion	2.26	0.98–5.21	0.053	Excluded*		
Neck stiffness	0.43	0.17–1.04	0.063	Excluded		
Limb weakness	2.94	1.49–5.78	0.001	Excluded		
Illness day (by +1 day later)	1.03	0.94–1.13	0.48	Not included±		
History of convulsion	0.75	0.36–1.54	0.44	Not included		
Rural origin	0.86	0.42–1.74	0.68	Not included		
Gender (female)	1.23	0.63–2.40	0.53	Not included		

NOTE:

# Results based on logistic regression;

*variables excluded after backwards variable selection;

**±:** variables not included in backwards variable selection.

## Discussion

Acute encephalitis is an inflammation of the brain parenchyma, most commonly caused by viruses and associated with substantial morbidity and mortality. Worldwide, reported mortality ranges between 0–11% [3], [4], [13]–[17], but was substantially higher in our study: 30% of children died during hospitalization, with about half of deaths occurring within 3–7 days after the onset of illness, and more than half affecting infants. Furthermore, 25% of surviving children suffered from mild to severe neurological sequelae at discharge (including: severe sequelae in 10%, moderate in 10% and mild in 5%). Several prognostic factors for death or severe outcome of acute encephalitis have been proposed [14], [15]. Whereas, similar to other studies, univariate analyses in our study suggested associations between fatal outcome and age, convulsions at admission and limb weakness, GCS and age remained the only independent prognostic factors for fatal outcome in logistic regression analyses.

Our study illustrates the challenge of identifying the causative agents in children with acute encephalitis. A confirmed or probable etiology was identified in only 41% of enrolled patients which is within the same range as reported in other etiology studies [3], [4], [13], [14], [18]. Worldwide, the causes of encephalitis vary between geographical regions. While JEV was the most common cause of encephalitis in children in Cambodia, enteroviruses and Tick-borne encephalitis virus were the two most common viruses found in young patients in the United States and Sweden, respectively [3], [4], [19]. A study in China revealed enteroviruses, mumps virus and rubella virus as frequent causes of encephalitis in children between 7 months and 13 years of age [20]. Our study identified JEV, enteroviruses and DENV as the most common etiologies in southern Vietnamese children, accounting for 26%, 9.3%, and 4.6% of cases, respectively. The high prevalence of JEV in our patients is in accordance with a previous study in Ho Chi Minh City reporting a prevalence of 45% in children with encephalitis [5]. Similar to previous studies, JEV was associated with high mortality (16%) [5], [21], [22]. Our observations emphasize the need for JE vaccination programs in Vietnam and other regions of Southeast Asia where JEV is endemic [4]. Widespread JE vaccination in developing countries like Vietnam is complicated by high costs and the requirement of multiple vaccine doses. In our study population, only 19.5% of patients had received at least one dose of JE vaccine. There was a trend of lower vaccination rates in JE patients, but this did not reach statistical significance (data not shown).

Enteroviruses are well established causes of aseptic meningitis and encephalitis in young children. While reported case fatality rates of enteroviral central nervous system (CNS) infections are relatively low (0–7%) [3], [13], mortality of confirmed or probable enteroviral encephalitis in our patients was high with 9 of 18 (50%) of patients dying during hospitalisation. However, a definitive diagnosis of enteroviral encephalitis was only established in 4 of these 18 patients, 2 of whom died. In the remaining patients enteroviral encephalitis was not confirmed by virus detection in CSF but suspected based on virus detection in throat and rectum and a clinical syndrome consistent with the diagnosis. Hence, a definitive causative role of enteroviruses in these patients remains unclear, particularly since a convincing link has not yet to be established between detection of enteroviruses in non-CNS sites and encephalitis [23]. In addition, beside enterovirus 71 (EV71), we have not determined whether our patients were infected with enterovirus serotypes of particularly high virulence, such as coxsackievirus B4 and echovirus 11 which have been associated with high case-fatality rates among neonates [24]. Six of 18 (33%) enteroviral infections in our patient group were caused by EV71. In accordance with reported high mortality of EV71 CNS infections, 3 of these patients died [25].

CNS manifestations are rare clinical complications of dengue but are reported with an increasing frequency in endemic areas [26]–[28]. Dengue was found in 4% of 378 pediatric patients with suspected encephalitis in southern Vietnam [27]. Similarly, DENV was identified in nearly 5% of our study patients. The precise pathogenesis of dengue-associated CNS manifestations remains unclear [27], [29]. Patients in this study experienced mild clinical symptoms of dengue without shock or bleeding and all made a full recovery. However, the detection of virus and specific antibodies in CSF suggest that invasion and viral replication in the CNS may play a role in at least a proportion of patients with dengue-associated neurological symptoms.

Me Tri virus is a variant of Semliki Forest virus belonging to the genus *Alphavirus* isolated from mosquitoes collected in Vietnam [10], [30]. Previous serologic surveillance suggested that this virus might be a frequent cause of encephalitis in young patients in Vietnam with a prevalence of 14% [30]. However, the virus was not detected in any of our patients. Likewise, human parechoviruses are an emerging cause of meningitis and encephalitis in children and infants [31], [32], but were not detected in our study patients. As a previous report suggested that human parechovirus infections exhibit a two-yearly incidence peak [33], the absence of parechovirus infections in our patients may be due to the fact that our study period covered only one year. HSV encephalitis was found in only one patient in our study which is not unexpected considering the young age of our study group.

Bacterial pathogens, including *S. pneumoniae* and *H. influenzae type B*, were detected retrospectively by PCR in 12 children (6%), illustrating the difficulties in distinguishing acute viral encephalitis and bacterial meningitis in children based on clinical judgment only. Indeed, the clinical syndromes of these children seemed indistinguishable from the remaining group of patients, although CSF white cell counts were higher as expected (Table 3). Of note, 4 children with bacterial meningitis had evidence of concurrent viral encephalitis based on the detection of virus (EV, CMV) or specific IgM (JEV) in the CSF. These observations suggest that testing for other pathogens should still be considered when a single pathogen has been identified in the CSF. While the clinical relevance of these coinfections remain unclear, it is tempting to speculate about the interplay between viral and bacterial CNS infections, for example by facilitating entry of one or the other into the CNS compartment.

In 59% of the children, no confirmed or probable viral etiology could be identified. Several factors may contribute to this relatively low diagnostic yield.

Firstly, a proportion of children may not have suffered from viral or other infectious CNS illnesses. Other conditions that may have presented in this manner may include pretreated pyogenic meningitis, toxins, atypical bacteria, mycobacterial and other unknown or un-tested bacteria or viruses. As mentioned before, bacterial meningitis was in fact diagnosed retrospectively by PCR in 6% of enrolled children.

A reliable case definition for acute viral encephalitis would be helpful for triage and clinical management of patients, especially in the absence of diagnostic support. When retrospectively using a predefined case definition consisting of a) fever history of less than seven days, b) an alteration or reduction of consciousness (Glasgow coma scale≤14), c) at least one of the following symptoms or signs: seizures (excluding febrile convulsions, defined as a single convulsion lasting less than 15 minutes in patients between 6 months and 6 years of age), focal neurological signs, neck stiffness or cerebrospinal fluid (CSF) pleocytosis, d) no evidence of bacterial infection in CSF specimens, and e) no alternative diagnosis for the clinical syndrome during admission, the viral diagnostic yield only modestly increased from 41 to 45%, whereas definitive/probable laboratory diagnoses of viral CNS infections were established in 20 of 62 patients (32%) who did not meet the criteria of the case definition. Overall clinical outcome and patients characteristics were similar between the two groups even though there were slight differences in terms of patient origins, clinical outcome, history of fever, limb weakness, neck stiffness, percentage of patient with a GCS below 9 and CSF cell count (Table 3). The last five are more likely due biases toward the criteria of predefined case definition.

Diagnostic yield was similar when the criterion of a fever history of less than seven days and febrile convulsions were left out. Diagnostic yield was higher when CSF results were included into this case definition (White cell count <1000 10e6/L, CSF/blood glucose ratio >50%, protein <0.45 g/L, CSF lactate <4 mmol/L): 49% and 37% in patients that did or did not meet the case definition, respectively. However, two thirds of all patients did not meet the case definition. Clearly, further studies are needed to optimize case definitions for viral encephalitis.

The limited detection of viral pathogens in our patients may have been due to clearance of virus from CSF at the time of clinical presentation, as is the case for JEV [34]. This may also explain why, except for dengue virus, flaviviruses were not detected in any of our patients, including those with serologically confirmed JEV infections. The importance of early sampling for reliable diagnostics is suggested by findings in our study: most of the patients were admitted relatively late in the course of illness, possibly in part because of referral delays, and a trend was observed towards shorter illness duration at the time of sampling in patients with positive virus-specific PCRs. The association between earlier sampling time and PCR positivity was statistically significant when analysis was restricted to 132 patients fulfilling our predefined case definition of acute viral encephalitis (3 days versus 4 days respectively, *P* = 0.03).

As diagnostic assays of some viral pathogens are not available in our laboratory, we did not exhaustively look for all potential infectious causes of encephalitis in Vietnam. Thus various etiologies (e.g. measles virus, henipaviruses and Banna virus) may have been missed. In addition, undiagnosed encephalitis may be caused by as yet unknown human or zoonotic pathogens. As socio-economic, ecological and environmental factors in Southeast Asia may favor the emergence of novel zoonotic or vector-borne pathogens [35], the circulation of novel pathogens in Vietnam is probable. Therefore, efforts to identify novel or previously unrecognized pathogens in these undiagnosed patients are essential for future prevention and treatment strategies.

## Supporting Information

Alternative Language Abstract S1 Translation of the Abstract into Vietnamese by Le Van Tan
(0.03 MB DOC)

Checklist S1 STROBE checklist
(0.09 MB DOC)
