# Supplementary material for: Viral Etiology of Encephalitis in Children in Southern Vietnam: Results of a One-Year Prospective Descriptive Study
Source: PLoS Negl Trop Dis. 2010 Oct 26;4(10):e854. doi: 10.1371/journal.pntd.0000854 (PMC2964288; doi:10.1371/journal.pntd.0000854)
Supplement: Alternative Language Abstract S1 — Translation of the Abstract into Vietnamese by Le Van Tan (0.03 MB DOC) [file pntd.0000854.s001.doc]

**Tóm Tắt**

**Giới thiệu**

Bệnh viêm não là một vấn đề nghiêm trọng ở Việt Nam. Mặc dù thông tin về bệnh nguyên là cần thiết cho việc ngăn ngừa và điều trị, nhưng hiểu biết về vấn đề này vẫn còn khá hạn chế ở Việt Nam. Với lý do đó, năm 2004 chúng tôi thực hiện một nghiên cứu mô tả kéo dài trong thời gian một năm ở Bệnh viện nhi đồng một, là bệnh viện tuyến cuối của khu vực phía nam Việt Nam bao gồm cả Thành phố Hồ Chí Minh.

**Phương pháp và kết quả cơ bản**

Tất cả các bệnh nhân có tuổi dưới 16 với triệu chứng lâm sàng giống viêm não và nghi ngờ là do vi rút được mời tham gia nghiên cứu. Chẩn đoán tìm tác nhân vi rút bao gồm nuôi cấy vi rút, huyết thanh học và khuyếch đại gen (PCR). Tác nhân vi rút được chẩn đoán xác định hoặc nghi ngờ trong 41% của 194 bệnh nhân. Các vi rút được chẩn đoán bao gồm vi rút viêm não Nhật Bản (50 bệnh nhân, chiếm 26%), enterovirus (18 bệnh nhân, chiếm 9.3%), vi rút dengue (9 bệnh nhân, chiếm 4,6%), vi rút herpes simplex (1 bệnh nhân), cytomegalovirus (1 bệnh nhân), và vi rút cúm A (1 bệnh nhân). Có 57 bệnh nhân (chiếm 29%) tử vong. Nguy cơ tử vong được xác định có mối liên hệ độc lập với tuổi của bệnh nhân và chỉ số hôn mê (GCS) thăm khám lúc nhập viện.

**Kết luận**

Tỷ lệ tử vong của bệnh viêm não trẻ em ở khu vực phía nam Việt Nam là rất cao. Mặc dù tác nhân gây bệnh không tìm thấy ở phần lớn các bệnh nhân, kết quả của nghiên cứu này có thể đóng góp một phần vào những nghiên cứu về ngăn ngừa và điều trị bệnh viêm não ở Việt Nam trong tương lai. Việc xác định nguy cơ tử vong có liên hệ độc lập với độ tuổi bệnh nhân và chỉ số hôn mê có thể có ích đối với các bác sĩ trong việc chăm sóc và điều trị bệnh nhân.
